# Molecular basis of fluoride toxicities: Beyond benefits and implications in human disorders

**DOI:** 10.1016/j.gendis.2022.09.004

**Published:** 2022-09-21

**Authors:** Priyankar Pal, Niraj Kumar Jha, Debankur Pal, Saurabh Kumar Jha, Uttpal Anand, Abilash Valsala Gopalakrishnan, Abhijit Dey, Prabir Kumar Mukhopadhyay

**Affiliations:** a Department of Life Sciences, Presidency University, Kolkata, West Bengal 700073, India; b Department of Biotechnology, School of Engineering and Technology (SET), Sharda University, Greater Noida, Uttar Pradesh 201310, India; c Department of Biotechnology, School of Applied & Life Sciences (SALS), Uttaranchal University, Dehradun 248007, India; d Department of Biotechnology Engineering and Food Technology, Chandigarh University, Mohali 140413, India; e Department of Life Sciences, Ben-Gurion University of the Negev, Beer-Sheva 84105, Israel; f Department of Biomedical Sciences, School of Bio Sciences and Technology, Vellore Institute of Technology (VIT), Vellore, Tamil Nadu 632014, India

**Keywords:** Fluoride, Autophagy, Mitochondrial dysfunction, Signaling cascades, System disorders

## Abstract

Detrimental impacts of fluoride have become a global concern for several decades. Despite its beneficial role which is restricted only in skeletal tissues, deleterious effects are also observed in soft tissues and systems. The generation of enhanced oxidative stress is the commencement of excess fluoride exposure which may lead to cell death. Fluoride causes cell death through autophagy via Beclin 1 and mTOR signaling pathways. Beside these, several organ specific anomalies through different signaling pathways have been documented. Mitochondrial dysfunction, DNA damage, autophagy and apoptosis are the damaging outcomes in case of hepatic disorders. Urinary concentration defects and cell cycle arrest have been reported in renal tissues. Abnormal immune response has been characterized in the cardiac system. Cognitive dysfunction, neurodegenerative condition and learning impairment have also been observed. Altered steroidogenesis, gametogenic abnormalities, epigenetic alterations and birth defect are the major reprotoxic conclusions. Abnormal immune responses, altered immunogenic proliferation, differentiation as well as altered ratio of immune cells are well-defined anomalies in the immune system. Though the mechanistic approach of fluoride toxicity in physiological systems is common, it follows different signaling cascades. This review emphasizes diverse signaling pathways which are the targets of overexposed fluoride.

## Introduction

Fluorine is the most electronegative halogen element which subsists in the environment as fluoride ion. Fluoride exists in nature in combination with the other elements and these fluorinated compounds are the constituents of minerals in soils and rocks, which are the natural contributors of fluoride.^1^ Apart from natural sources, anthropogenic fluoride contamination occurs through industrialization, fluoride-containing pesticides, supply of fluorinated drinking water, refrigerants, dental products and fire extinguisher.^2^ The major source of the fluoride revelation to the human population is contaminated drinking water.^3^

Interestingly, fluoride exerts its action within the human body in a concentration-dependent manner which is unlike the other contaminants. Below 1 mg/L concentration it exhibits beneficial effects in terms of teeth and bone development; however high exposure which is beyond the WHO permissible upper limit (1.5 mg/L) leads to an adverse health condition known as fluorosis.^4^ About 200 million people around the globe from approximately 20 countries are at risk due to consumption of fluoride contaminated drinking water^5^ and in India, the number is about 62 million covering 177 districts belonging to 20 states.^3^

After ingestion fluoride is absorbed through the stomach and small intestine in a pH-dependent and independent manner respectively.^6^ In the acidic environment of the gastric lumen, fluoride is absorbed in the form of hydrogen fluoride while in the proximal small intestine fluoride is absorbed in the form of fluoride ion.^7^ The maximum amount of absorbed fluoride is retained in the calcified tissues but the distribution of fluoride to the soft tissues depends on the rate of the blood flow to those organs. The ratio of fluoride concentration in tissue/plasma varies among different tissues where kidney exhibits a high ratio compared to other organs.^8^ Urinary excretion is the major pathway of fluoride removal from the body.

Dental and skeletal fluorosis are the major destructive consequences of excess fluoride exposure; nevertheless, it has been recognized that soft tissues are also vulnerable.^9^ Being a biologically and chemically active component, fluoride can rapidly cross the biological membranes and put forth its toxicities via generation of oxidative stress. Fluoride toxicity is associated with overproduction of reactive oxygen species (ROS), generation of nitric oxide (NO) and also reduction of antioxidant defense. Enhanced ROS generation can be explained by the production of superoxide anions which in turn activates downstream consequences like the generation of peroxynitrite, hydrogen peroxide and hydroxyl radicals. NO reacts with superoxide anions forming peroxynitrite and also reacts with metal centers along with thiols of proteins forming nitrosyl adducts.^1^ All these products can generate oxidative stress. Excessive ROS production results in free radical attack of the membrane phospholipids and causes lipid peroxidation along with depolarization of mitochondrial membrane.^1^ Fluoride also causes DNA damage in different cell types due to upsurge oxidative strain.^10^ It also suppresses the activity of the DNA polymerase enzyme thereby affecting DNA replication and also repair process which also supports the evidence of fluoride mediated DNA damage.^11^ Available reports revealed that fluoride causes inhibition of protein synthesis and/or secretion and leads to activation of signaling pathways engaged in proliferation and also apoptosis which include nuclear factor kappa B (NF-κB), activator protein-1, mitogen-activated protein kinase (MAPK) and p53.^10^^,^^12^^,^^13^

Animal experimentations divulged the extent of fluoride toxicities in different soft tissues and systems mainly through morphological abnormalities, alteration of redox status, functional anomalies, autophagy, apoptosis and inflammatory disorders through different altered signaling cascades. Liver is responsible for the maintenance of metabolic homeostasis of the body and is susceptible to fluoride toxicity. It alters the balance of pro-oxidants and antioxidants of the liver which ultimately causes morphological, biochemical and functional abnormalities.^14^ Kidney is a soft target of fluoride-induced adverse effects due to metabolism kinetics, bio-concentration and excretion.^15^ Enhanced oxidative stress in terms of increased nitrosative stress and decreased antioxidants lead to damages of nucleic acid, protein as well as the structure–function aspect of nephron.^16^^,^^17^ Fluoride mediated enhanced oxidative strain also leads to cardiac disorders like ischemia and cardiac failure.^18^ Nicotinamide adenine dinucleotide phosphate oxidase (NOX) is a vital source of ROS in vasculature which is activated by excess fluoride and leads to vascular disorders and coronary heart disease.^19^ Fluoride is also considered as the potent toxin of the central nervous system which causes altered cerebral function, neuronal apoptosis, impairment of learning and memory.^20^^,^^21^ Structure–function alterations of testis and epididymis have been reported due to fluoride threat which gradually leads to male infertility.^22^ Decreased fertility, reduced number of viable fetuses and endometrial apoptosis have been well characterized in the female reproductive system following overexposure to fluoride.^23^ Inflammation is the first response of the immune system following tissue damage or infection and over-exposed fluoride is responsible for the generation of such response.^1^ Expression of the pro-inflammatory cytokines is under the control of transcription factors including NF-κB which also exerts fluoride mediated regulation.^10^

Although apoptosis and autophagy are the common disadvantageous conclusions upon fluoride contamination in most organs, modes of action and the other altered signaling pathways are distinct in different organs. There is meager information regarding different molecular cascades through which fluoride affects different soft tissues/organs.

Here we have tried to accumulate the molecular events which are distressed due to overexposed fluoride in hepatic, renal, cardiovascular, neuronal, reproductive and immune systems. Table 1 presents the various system disorders with altered genomic and/or proteomic expressions caused by fluoride.

**Table 1:** Various system disorders with altered genomic and/or proteomic expression caused by fluoride.

System disorders	Altered genomic and/or proteomic expression	Extent of damages	Reference
**Hepatic**	↓CuZn-SOD, Mn-SOD, GST, CAT and GSH-Px; ↓NDUVF2; ↑SDHA and CYC1; ↑OPA1 and Mfn1; ↑Dyn2, Drp1, Fis1, Mff, MiD49, and MiD51; ↓RPS3; ↑Beclin-1 and LC3; ↓ p62; ↑Cyt-c, Apaf-1, caspase3, caspase12, p53, IL-17, IL-17*r*, Act1, NF-kB, IkB, and p65; ↓Bcl-2; ↑TNF-R1, FADD, TRADD, caspase-8, caspase-3 and c-Fos; ↓Dnmt1, Dnmt3a and Dnmt3b	Oxidative stress; Damaging of mitochondrial respiratory chain complex; Altered energy metabolism; Abnormal mitochondrial fission and DNA damage; Autophagy; Apoptosis	*27*, *34*, *38*, *39*, *41*, *43*, *47*
**Renal**	↓SOD2, Nrf2; ↑ Kim-1, NF-kB, IL-6 and TNF-α; ↓Cyclin B1, CDK1 and mdm2, Cdc25C and PCNA; ↑ATM, Chk2, p53, p21 and Gadd45a; ↑Bax, Bak and cytosolic Cyt-c, p-JNK1/2, p-c-Jun, TNF-a, and Bak; ↓ Bcl-2; ↓Ca2+-ATPase; ↑LTCC Cav1.2 and Bax/Bcl-2; ↓Na–K-ATPase pump	↑ROS, NO and oxidative stress; Cell cycle arrest; Apoptosis; Urinary concentrating defects	*54*, *56*, *60*, *69*, *70*, *72*, *73*
**Cardiac**	↑Phosphorylation of AMPKα, AMPKβ1, ACC and Nox4; ↓ATP; ↑Bax, TNF- α, Fas, caspase −8, Cyt-C, caspase-9, caspase-3 and P38αMAP kinase; ↓Bcl2; ↓IL-1, IL-6 and IL-10;	↑ROS and oxidative stress; Apoptosis; Altered immune response	*74*, *78*, *79*, *80*, *81*
**Neuronal**	↓PLP, CREB and BDNF; ↓ Keap1; ↑Nrf2, GST, CAT, *Nqo1*and *p38*; ↑Calpain-1, GTPaseRhoA; ↓ERK1/2, BDNF and CREB; ↓Synaptophysin, MAP2, Dbn, NMDAR, IR and mGluR5; ↓α4 and α7 subunits of nAChR; ↓ PSD-95 and SYN; ↑p-ERK ½; ↓NCAM; ↑OX-42 positive cells, TNF-α, IL-1β, and IL-6; ↑GSK-3β, β-catenin, cyclin-D1 and c- myc; ↓Tubα1a and Tubβ2a; ↓GFAP and GLUT1; ↑BDNF; ↑Mfn1 and Mfn2; ↓ Drp1 and Fis1; ↓Mfn1; ↑ Drp1 and Fis1; ↓PGC-1α and TFAM; ↑Beclin-1, lipofuscin, Bax; ↓Bcl2; ↓LC3-II and Atg5; ↑p62, mTOR and p70^S6K^; ↑Beclin1, LC3-II and p62; ↑IRE1α, GRP78; ↑ p53, Fas, Fas-L, caspase 8 and caspase 3	Altered synaptic structure and myelin damage in hippocampus; Redox imbalance; ↓Cognition, ↓Learning and memory; ↓Synaptogenesis; ↓brain function, ↑Neurodegeneration; ↑Brain atrophy, interstitial neuronal edema; Altered mitochondrial dynamics and brain injury; Altered mitochondrial biogenesis and cell death; Defective autophagy, ER stress	*83*, *85*, *91*, *92*, *93*, *95*, *96*, *97*, *98*, *101*, *102*, *103*, *104*, *105*, *106*, *107*, *108*, *112*, *113*, *114*
**Male reproductive**	↓Nrf2, HO-1, g-GGL, NQO; ↑Kepa 1; ↓GST, GPx and GR; ↓Cyp11a1, Star, Hsd3b1, Hsd17b3, Gata-4, Sf-1 and Nur77; ↑Dax-1; ↓*INH*α, INHβB, FSHR, LHR and SHBG; ↑piR-mmu-1566,415, piR-mmu-8060,747, piR-mmu-1277,316, Gga2, Ap4e1, Ap1s3 and Gla; ↑NF-κB, TNF-α, IL-1β, iNOS and COX-2; ↑PINK1 and PHB2; ↑caspase −3 and Fas, chromatin condensation, DNA fragmentation and cytochrome C; ↑Caspase-3, Caspase-9 and Bax; ↓Bcl-2; ↑GRP78, PERK, p-eIF2α and CHOP; ↑Beclin 1, Atg5 and LC3 II; ↓Phosphorylation of mTOR; ↓ATK; ↑LC3 II and p62; ↓DDX25, TP2, H4, PGK2 and HMG2; ↓P450; ↑CREM and ACT; ↓Tyrosine phosphorylation, PKA activation; ↓CatSper 1; ↓mt-Cytb and mt-COX2; ↓NGF, Ras, Raf, and MEK; ↓ZPBP1, SPACA1 and DPY19L2 and LMNB2; Altered expression of AKAP3, AKAP4, CFAP43, CFAP44 and HYDIN; ↓Sly, Ssty2 and HSF2; Altered expression of ACR, PRSS21, SPAM1, CD9 and CD81; ↑Ki-67 and PCNA	Oxidative stress; Altered steroidogenesis; Altered lysosomal degradation pathway; Testicular inflammation and Mitophagy of Leydig cell; Apoptosis; ER stress and unfolded protein response; Autophagy; ↓Spermatogenesis; ↓Capacitation, acrosome reaction and chemotaxis of spermatozoa; ↓Sperm motility; Altered ultrastructure of nuclear lamina and acrosome; Altered fibrous sheath and axonemal structure of sperm flagellum; Sperm head abnormalities	*115*, *120*, *121*, *122*, *123*, *124*, *126*, *127*, *128*, *129*, *130*, *131*,*134*, *135*,*138*, *139*, *141*, *142*, *143*
**Female reproductive**	↓SOD1, GSH-Px1 and CAT; ↑Estrogen and progesterone receptor; ↓FSHR; ↑ Mfn1 and OPA1; ↑NDUFV2, SDHA and CYC; ↑miR-29 b; ↓DAZL, Nobox, Sohlh1, and Zp3; ↓Bmp15, Gdf-9, zp2 and H1oo; ↓NNAT; ↓JNK; ↑STAT3, STAT5, CDK2 and CDK4; ↑PCNA; ↑Bax, Caspase-3 and Caspase-9; ↑MMP-9 and TIMP-1; ↑H3K9m2 and H3K4m2	Redox imbalance; Altered steroidogenesis; Abnormality in mitochondrial fusion, Mitochondrial dysfunction; ↓Formation and fertilization of mature oocytes; ↓Oocytes maturation and quality; DNA damage, Apoptosis and follicular dysplasia in ovary; ↓Litter size; Epigenetic modification	*25*, *148*, *149*, *151*, *152*, *153*, *155*, *159*, *160*
**Immune**	↓H3K18ac and H3K9ac; ↑BiP, GRP94, P-IRE1, P-PERK, ATF6, P-eIF2a and ATF4; ↓IL-2, IFN-γ and T-bet; ↑IL-4, Il-6 and GATA3; ↓Cyclin D, cyclin E, CDK2 and CDK4; ↓Foxn1, Cbx4, DLL4, IL-17, CD2, PTPRC, CD69 and CD101; ↓IgA, IgM and IgG; ↑IL-2, IL-6, TNF-α, IFN-γ and TGF-β; ↓TLR2/MyD88, IRAK1, IRAK4, TAK1, TRAF6 and c-JUN; ↑CHOP, p-JNK, Cleaved-caspase12, Bax and Bak, Fas, FasL, caspase 9, caspase 8, caspase 7, caspase 6 and caspase 3; ↓Bcl-2 and Bcl-xL; ↑LC3, Beclin1, Atg16L1, Atg5 and Atg12; ↓p62; ↓PI3 and AKT	↓Germline line development; ER stress in spleen; Altered ratio of Th1/Th2; ↓Proliferation of splenic lymphocytes; Altered T cell proliferation and differentiation; ↓Immune response; ↓Innate immunity; Apoptosis; Autophagy; ↓mTOR signaling	*152*, *161*, *167*, *168*, *170*, *172*, *175*, *185*

### Fluoride toxicity onset

The generation of oxidative stress is the key mediator involving fluoride-induced tissue damages. Aside this, fluoride also initiates some other detrimental pathways. It disrupts Na^+^/−K^+^/−ATPase and leads to hyperkalcemia as well as decreased activity of acetylcholinesterase which eventually cause vomiting, diarrhea and hypersalivation.^15^ It also interrupts glycolysis, oxidative phosphorylation, neurotransmission and coagulation. Fluoride causes membrane damage of the endoplasmic reticulum and leads to activation of stress signaling. Disruption of Ca^2+^ homeostasis in the ER has also been reported due to over-exposed fluoride and it has been hypothesized that ER stress is associated with oxidative stress.^24^ The damaging effects are also observed in mitochondria which include loss of membrane integrity and inhibition of the activities of respiratory enzyme complexes.^25^ Fluoride can also combine with DNA forming a covalent bond and thus affects DNA strands directly and also indirectly through free radicals generation.^26^

Signaling pathways targeted by fluoride in different systems are briefly presented here.

### Hepatic disorders

**Figure 1:**
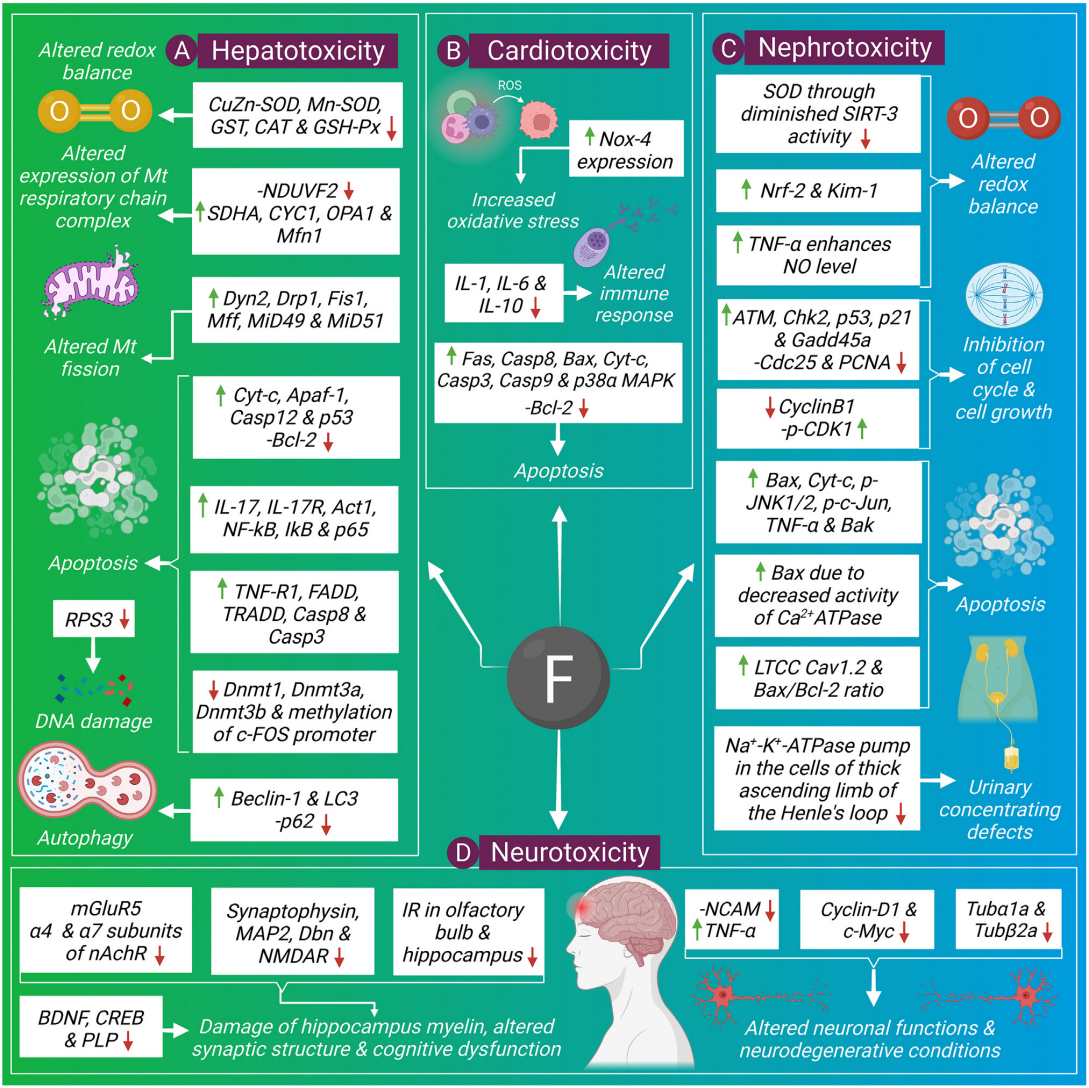
Signaling pathways of fluoride induced hepatotoxicity **(A)**, cardiotoxicity **(B)**, nephrotoxicity **(C)**, and neurotoxicity **(D)**.

Liver is the fundamental metabolic organ responsible for oxidative detoxification of xenobiotic compounds and any disorder in the liver leads to difficulties of biological systems. The key hepatic disorder is alteration of metabolic pathways which are related to energy metabolism.^27^ Excess fluoride affects hepatic proteins which are associated with energy metabolism as well as altered mitochondrial functions.^28^ Fluoride mediated morphological changes in hepatic tissues have been reported which include hepatic hyperplasia, necrosis, vacuolization, fatty changes, dilated central vein and hepatocytic drop off.^29^, ^30^, ^31^, ^32^ Fluoride is liable for enhanced lipid peroxidation and generation of oxidative stress through reducing hepatic antioxidant defense.^33^ Activities of hepatic functional enzymes also become altered due to over-exposure to fluoride.^33^ The associated events liable for alteration of hepatic functions are illustrated here **(**Fig. 1 A).

### Redox imbalance

Superoxide dismutase (SOD), catalase (CAT), glutathione peroxidase (GSH-Px) and glutathione transferase (GST) are important enzymes which eliminate ROS. Fluoride decreases the mRNA expression of hepatic CuZn-SOD (copper, zinc SOD), Mn-SOD (manganese SOD), GST, CAT and GSH-Px^34^ thereby provoking the oxidative stress.

### Mitochondrial dysfunction

#### Effects on mitochondrial respiratory chain and energy metabolism

Mitochondrial respiratory chain consists of four multi-subunit complexes (I, II, III and IV) which are embedded in the inner mitochondrial membrane and responsible for ATP generation through oxidative phosphorylation.^35^ A subunit of complex I is NADH dehydrogenase [ubiquinone] flavoprotein 2 **(**NDUVF2) and loss of its function is associated with lipid peroxidation, ROS generation, mitochondrial dysfunction and diminished ATP production.^36^ The succinate dehydrogenase complex flavoprotein subunit A (SDHA) and cytochrome c1 (CYC1) are subunits of complex II and III responsible for ATP production in eukaryotic cells.^37^ Fluoride down-regulates the mRNA and protein expression of NDUVF2 while up-regulates the expression of SDHA and CYC1 in the liver, thereby damaging the mitochondrial respiratory chain.^38^ Mitochondrial fusion proteins include optic atrophy 1 (OPA1) and mitofusin-1 (Mfn1), which are regulatory factors of membrane fusion remodeling and mitochondrial cristae morphology; alteration of these protein expression affect cellular ultrastructure and mitochondrial distribution.^39^ Fluoride also up-regulates the mRNA and protein expression of OPA1 and Mfn1 in the liver which affects energy metabolism.^38^

So it can be concluded that fluoride-induced hepatic damages follow mitochondrial injury through altered expression of proteins in respiratory chain complexes.^38^

#### Effect on mitochondrial fission

The dynamin-related protein 1 (Drp1)/mitochondrial fission factor (Mff) signaling pathway has a pivotal role in mitochondrial fission. An affiliation is created between mitochondria and endoplasmic reticulum with the help of actin filaments through inverted formin 2 (INF2), which in turn promotes the synthesis of mtDNA and recruits Drp1 protein. Subsequently, Mff, fission protein 1 (Fis1), mitochondrial elongation factor 1 (MIEF1/MiD51) and mitochondrial elongation factor 2 (MIEF2/MiD49) recruit Drp1 from the cytoplasm and endoplasmic reticulum to mitochondrial surface. Lastly, dynamin-2 (Dyn2) acts together with Drp1 and maintains mitochondrial constriction as well as completes the fission process.^40^ Over-expressed Dyn2 and Drp1 exhibit structural damages of mitochondria. On the other hand, enhanced expressions of Fis1, Mff, MiD49, and MiD51 cause fragmentation of mitochondria. Fluoride up-regulates the mRNA and protein expression of these genes in mitochondria of hepatocytes and causes abnormal mitochondrial fission.^41^

### DNA damage

A part of the 40 S ribosomal subunit is the 40 S ribosomal protein S3 (RPS3) and when it is present inside the mitochondria, it is capable to reduce ROS levels and in turn diminishing the DNA damage.^42^ Fluoride has been known to reduce the expression of this protein and indicates a poor defense against ROS and enhanced DNA damage.^27^

### Autophagy

Two important autophagy markers are Beclin-1 and microtubule-associated protein 1 A/1 B-light chain 3 (LC3), both of which promote autophagosome formation. Another marker is p62, which has a negative correlation with autophagy. Fluoride increases the protein expression of Beclin-1 and LC3 while decreases the expression of p62 in hepatocytes, suggesting that it promotes autophagy in the liver.^43^

### Apoptosis

#### Cytochrome C mediated pathway

Fluoride has been known to provoke apoptotic pathway in the liver. Key regulators of apoptosis are Bcl-2, cytochrome C (Cyt-c), apoptotic protease activating factor 1 (Apaf-1), caspase-3, caspase-12, and p53; among these Bcl-2 is anti-apoptotic protein and rest are pro-apoptotic. Fluoride enhances mRNA expression of Cyt-c, Apaf-1, caspase-3, caspase-12, and p53 but decreases the mRNA expression of Bcl-2 in the liver. Protein expression of caspase-3 and Cyt-c were also increased like mRNA levels.^43^ Therefore, apoptosis is one mode of fluoride mediated hepatic damage.

#### NF-kB mediated pathway

Interleukin-17 (IL-17) is a pro-inflammatory mediator, secreted by helper T cell Th17, which is a subset of CD4^+^ Th cells. Increased level of IL-17 was observed in several liver lesions like liver fibrosis, fatty liver, hepatitis etc.^1^ IL-17 functions by binding to its receptor IL-17*r*, which then activate the formation of IL-17 R–Act1–TRAF6 complex and this complex then activates nuclear factor kappa light chain enhancer of activated B cells (NF-κB) signaling pathway that causes tissue damages.^44^ Fluoride up-regulates the mRNA expression of IL-17, IL-17*r*, NF-κB activator 1 (Act1), NF-κB, an inhibitor of nuclear factor kappa B (IκB), and p65 which are all IL-17 signaling pathway associated genes. It can be concluded that fluoride promotes hepatic damage and apoptosis by the IL-17 signaling pathway.^43^

#### TNF-R1 mediated pathway

Tumor necrosis factor receptor (TNFR) family has two receptors, among which TNF-R1 is involved in apoptosis.^45^ After binding of ligand to the receptor, this complex recruits adaptor proteins Fas-associated death domain (FADD) and TNFR-associated death domain (TRADD) and ultimately form death-inducing signaling complex (DISC), which activates pro-apoptotic proteins caspase-8 and caspase-3. Fluoride increases mRNA expression and proteins level of TNF-R1, FADD, TRADD, caspase-8 and caspase-3 in hepatic tissues.^34^ This study depicts that fluoride follows the TNF-R1 signaling pathway to promote hepatocellular apoptosis.

#### c-Fos mediated pathway

One proto-oncogene is c-fos which encodes transcription factor responsible for cellular proliferation, differentiation as well as apoptosis. Xenobiotic studies revealed that altered DNA methylation affects cellular transformation and an in-depth study demonstrated that the CpG island of the c-Fos promoter is generally hypermethylated in normal liver.^46^ DNA methyltransferases (Dnmts) catalyze methyl group transfer in the CpG, producing 5-methyl cytosine and Dnmt1 is the best characterized Dnmt. It catalyzes methylation of the newly synthesized DNA strand by copying the parental strand, while Dnmt3a and Dnmt3b are responsible for de novo methylation at the new location of the DNA strand. Fluoride down-regulates the mRNA expressions of Dnmt1, Dnmt3a and Dnmt3b in the human embryo hepatocyte (L-02), thereby reducing the methylation pattern of the c-Fos promoter. This demethylation, in turn, up-regulates c-Fos expression, which leads to apoptosis of L-02 cells.^47^

### Renal disorders

Urinary excretion is the key event through which fluoride level is regulated in the body. The renal system is also vulnerable to fluoride toxicity. Structural deformities of the renal system with over-exposed fluoride have been well characterized by the shrunken and lobulated appearance of a glomerulus, dilated renal tubule, infiltration of inflammatory cells in renal tissues, vacuolar degeneration of tubular epithelium as well as structural alterations of proximal, distal and collecting tubules.^31^^,^^48^ Fluoride also alters redox homeostasis in renal tissues in terms of increased lipid peroxidation and decreased antioxidants activities.^49^ Fluoride mediated altered renal functions evidenced by an increased level of urea, creatinine, Na^+^ and K^+^ indicate the inability of the kidney to remove toxic substances.^50^ Here we have discussed molecular detail concerning fluoride mediated renal damages (Fig. 1C).

### Redox imbalance

#### SIRT3 mediated pathway

One key mitochondrial deacetylase is sirtuin3 (SIRT3), which modulates several proteins for control of mROS level.^51^ Residing primarily in mitochondria, SIRT3 promotes forkhead box O (FoxO3a) translocation to the nucleus and activates FoxO3a dependent SOD2 gene.^52^ SOD2 is a substrate of SIRT3 and binding of these two results in deacetylation and activation of SOD2.^53^ Fluoride reduces the expression of SOD2 through SIRT3 dependent DNA binding activity of FoxO3a and inhibits the activity of SOD2 by diminishing SIRT3 mediated deacetylation and causing oxidative damage.^54^

#### Nrf2 mediated pathway

Nuclear factor erythroid 2 p45 (NF-E2)-related factor (Nrf2) plays an important role inthe regulation of antioxidant enzymes and induces the expression of several genes directly or indirectly. Thus activated Nrf2 is an important molecular target of several chemoprotective agents.^55^ Fluoride decreases the expression of Nrf2 in renal tissues^56^ and enhances oxidative stress.

#### Kim-1 mediated pathway

Kidney injury molecule-1 (Kim-1), also known as hepatitis A virus cellular receptor 1 (Havcr1in), which is a type I transmembrane protein and not detected in normal kidneys but highly expressed after ischemic or toxic injury.^57^ Fluoride enhances the expression of Kim-1 in the proximal tubule of the rat kidney. This study revealed that increased ROS production by fluoride intoxication results in kidney injury in the proximal convoluted tubule.^56^

#### Pro-inflammatory cytokines and interleukin mediated pathway

The levels of NF-kB, IL-6 and tumor necrosis factor-α (TNF-α) become elevated upon fluoride intoxication. NADPH oxidase (Nox) generates ROS along with pro-inflammatory cytokines and that are the mediators of fluoride-induced oxidative stress generation and inflammation.^58^ TNF-α is a pro-inflammatory cytokine, which triggers several interleukins such as IL-1, IL-6 and IL-8. TNF-α also up-regulates the expression of inducible nitric oxide synthase (iNOS), which in turn produces nitric oxide (NO) and excess NO produces peroxynitrite radical after reacting with superoxide anion and subsequently causes cellular damages of renal tissues.^56^

### Altered cell cycle and cell growth

#### Cyclin and CDK mediated pathway

Cell-cycle is a multipart event responsible for growth and proliferation of cells and it can be divided into four different stages such as G1, S, G2 and M. Cell cycle checkpoint controls transitional steps of each stage of cell cycle i.e., the progression of the cell cycle. The checkpoint of the G2/M phase is under control of Cyclin B1/CDK1 complex and this complex remains inactive by diminution of Cyclin B1 protein and/or phosphorylation of CDK1 (Tyr-15).^59^ Fluoride declines the mRNA expression of Cyclin B1 along with CDK1 and the protein expression of CyclinB1 also becomes decreased. These studies signify that fluoride promotes renal damage by inhibiting the cell cycle at the G2/M phase.^60^

#### ATM-Chk2-p53 mediated pathway

Appropriate transition of G2/M phase is under the regulation of Cyclin B1/CDK complex.^61^ The previous report suggested that fluoride causes DNA damage in the kidney^62^ and DNA damage occurs after the activation of the ataxia-telangiectasia mutated (ATM) signaling pathway.^63^ Fluoride activates the ATM pathway by enhancing ATM mRNA expression level and also elevates the protein level of p-ATM (Ser-1981).^60^ Activated ATM recruits at the site of DNA damage and activates downstream signaling proteins by phosphorylation of checkpoint kinase 2 (Chk2) and p53.^64^ Fluoride increases the mRNA expression of both genes and as well as protein level of p-Chk2 (Thr-68) and p-p53 (Ser-15).^60^ Murine double minute 2 (Mdm2) is another protein, which binds with p53 and suppresses its activity by proteolytic degradation.^63^ Fluoride decreases the mRNA and protein level of mdm2 and further activates and accumulates p53.^60^ Activated p53 promotes the transcription of downstream effector genes such as p21 and Gadd45a. Fluoride up-regulates both mRNA and protein expression of these genes.^60^ These studies revealed that fluoride causes renal damage by inhibiting the growth and proliferation of renal cells through the ATM-Chk2-p53 signaling pathway.

#### ATM-Chk2-Cdc25 mediated pathway

Besides p53, the activated form of Chk2 inhibits the activity of CDK1 by phosphorylating a protein, Cdc25.^65^ Cdc25 dephosphorylates CDK1 and promotes cell division by activating it. It has been reported that fluoride decreases mRNA expression of Cdc25C and also diminishes the protein level of p-Cdc25C (Ser-216), which suggest that fluoride inhibits cell division and proliferation by following the ATM-Chk2-Cdc25 signaling pathway.^60^

#### Effect on G2/M phase

The previous report demonstrated that p21 inhibits DNA synthesis after binding and inhibiting proliferating cell nuclear antigen (PCNA), which acts as a processivity factor of DNA polymerase.^66^ Fluoride inhibits mRNA and protein expression of PCNA in renal tissues, thereby proliferation and development of renal cells are suppressed and this effect might be due to cell cycle arrest in renal tissues at the G2/M phase.^60^

### Apoptosis

#### JNK signaling pathway

Jun N-terminal kinases (JNKs) are protein serine/threonine kinases and belong to a superfamily of MAP-kinase, which are activated by oxidative stress and promote apoptotic signaling pathways by both extrinsic and intrinsic mode of action.^67^ After combination with ROS, JNK provokes apoptosis by enhancing the activities of the pro-apoptotic proteins and diminishing the activities of the anti-apoptotic proteins.^68^ Fluoride enhances the expression of pro-apoptotic proteins like Bax, Bak and cytosolic Cyt-c and reduces the expression of anti-apoptotic protein Bcl-2 in renal tissues, which propose that exposure of fluoride promotes renal injury by JNK signaling pathway followed by mitochondrial apoptotic pathway.^69^

JNK signaling pathway also engaged in extrinsic apoptotic pathway and the previous report demonstrated that JNK-c-Jun/Ap1 pathway enhances the expression of several pro-apoptotic genes like TNF-a, Fas-L and Bak.^67^ Fluoride up-regulates the expression of p-JNK1/2, p-c-Jun, TNF-a, and Bak in renal tissues, which states that fluoride promotes apoptosis via the JNK signaling pathway through the extrinsic method.^69^

#### Ca^2+^ ATPase dependent pathway

Elevated level of Ca^2+^ ATPase activity is a key regulator, which protects cells against abnormal high calcium accumulation. Over-exposed fluoride increases intracellular calcium concentration as well as decreases the activity of Ca^2+^ ATPase. Excess fluoride enhances apoptosis through up-regulating pro-apoptotic gene (Bax), which might be closely associated with an inhibitory effect of fluoride on Ca^2+^ ATPase activity along with augmented intracellular calcium level.^70^

#### LTCC mediated pathway

L-type calcium channel (LTCC) is an important route of calcium influx which also regulates intracellular calcium level.^71^ Fluoride up-regulates LTCC Cav1.2 in renal cells; thus, enhancing extracellular calcium uptake and leading to calcium overload within the cells. Increased intracellular calcium causes abnormal expression of the downstream molecules like CaM and CAMKII as well as enhances Bax/Bcl-2 ratio which ultimately leads to apoptosis.^72^

### Urinary concentrating defects

#### Altered activity of Na–K-ATPase pump

The Na–K–2Cl co-transport and Na–K-ATPase pump are responsible for potassium ion influx in renal cells. Fluoride exerts its toxic effects in the cells of the thick ascending limb of Henle's loop which include a decreased activity of the Na–K-ATPase pump. This suggests that the Na–K-ATPase pump is a target of fluoride toxicity which is in turn responsible for urinary concentrating defects.^73^

### Cardiac disorders

Excess fluoride accumulation also causes cardiac damage in several aspects. Fluoride mediated pathological alterations include structural deformities of myocardium in terms of the altered arrangement of the myofilaments, gaps between the myocardial fibers, breakage of myocardial fibers, fracture of Z and M lines, rupture as well as vacuolization of mitochondria.^74^ Cardiac tissues are also damaged by free radical attack due to over-exposed fluoride as evidenced by increased lipid peroxidation and diminished antioxidant defense.^75^ Fluoride also alters the expression of functional markers such as lactate dehydrogenase, creatine kinase-MB, aspartate aminotransferase, alanine aminotransferase, troponin T and troponin I which leads to necrotic damages of myocardial tissues.^75^ Fluoride mediated altered signaling pathways involving myocardial disorders are described here (Fig. 1B).

### Redox imbalance

#### Altered AMPK signaling pathway

An energy homeostasis regulator of higher eukaryotes is AMP-activated protein kinase (AMPK) and activation of this signaling molecule occurs mainly during enhanced energy deprivation, increased ratio of intracellular AMP and ADP in the cardio pathological condition in humans and other animals.^76^ In cardiotoxic conditions increased ROS production is an indicator of activated AMPK signaling molecule.^77^ Fluoride increases phosphorylation of AMPKα (Thr 172), AMPKβ1 (Ser 108) and ACC (Ser79) in the myocardium and causes decreased level of myocardial ATP and ultimately energy depletion.^78^ So, altered AMPK expression in cardiomyocytes may be due to fluoride threat.

#### Nox4 mediated oxidative stress

Nox4 is a member of the NADPH oxidase family and is widely distributed in the mitochondria of cardiomyocytes, overexpression of which is responsible for deleterious effects like oxidative stress and apoptosis. Fluoride increases Nox4 expression in the myocardium.^79^ Thus, Nox4 induced oxidative stress generation is a possible mechanism for fluoride-induced cardiotoxicity.

### Apoptosis

#### Altered expression of Bax and Bcl2

Fluoride hampers the cardiovascular system through different mechanisms of action, among which Bax and Bcl-2 signaling pathways are important mechanisms of action through which fluoride causes myocardial apoptosis. An experimental study revealed that expression of Bax protein is increased while expression of Bcl-2 protein is decreased after fluoride intoxication in a dose and time-dependent manner.^80^ So fluoride disrupts normal myocardial functions through this apoptotic pathway.

#### Extrinsic and intrinsic pathways

TNF-α is responsible for myocardial dysfunction and promotes cardiac injury, inflammation and apoptosis. Fluoride increases the expression of TNF-α, which promotes cardiac inflammation. Fas protein belongs to the tumor necrosis factor family and after combining, with Fas ligand it activates FADD, caspase-8 and caspase-3, which lead to apoptosis. Fluoride also increases the expressions of Fas and caspase-8. This study also reported that fluoride treatment causes oxidative stress and damages the mitochondrial membrane of the heart and promotes the release of Cyt-C, which further activates caspase-9 and caspase-3 and enhance apoptotic cell death.^81^

#### MAP kinase pathway

A member of the MAP kinase family is P38α MAP kinase which expresses predominantly in the heart and shows pro-apoptotic activities.^82^ Fluoride increases the expression of P38α MAP kinase in the myocardium. So fluoride mediated up-regulation of P38α MAP kinase is responsible for apoptotic damages in the myocardium.^79^

### Altered immune response

#### Abnormal levels of cytokines

Toll-like signaling molecules like IL-1, IL-6 and IL-10 are associated with immune response. Fluoride decreases both genomic and proteomic level of such cytokines in cardiomyocytes.^74^ Therefore, it can be concluded that fluoride affects the immune response of cardiac tissues by affecting cytokines.

### Neuronal disorders

Fluoride is able to cross the blood–brain barrier and results in neurotoxicity. Experimental studies on animal models revealed several neurodegenerative diseases. Structural disintegrations have been characterized by the disappearance of mitochondrial cristae, swollen endoplasmic reticulum, mitochondrial vacuole formation, decreased Nissl body density in the hippocampus and demyelination of the nerve fibers.^83^, ^84^, ^85^, ^86^ Fluoride also causes synaptic impairment due to provoked oxidative stress.^87^ Hippocampus is the region which plays important role in processing and integrating the external information and distributing the information to other cortical regions. As compared to other regions of the brain it is more susceptible to internal and as well as external oxidation.^88^ Signaling pathways of fluoride mediated neurotoxicity are briefly discussed here (Fig. 1D).

### Structural alterations

The structural integrity of the myelin sheath is associated with proteolipid protein (PLP). Fluoride decreases the mRNA expression of PLP, which indicates damage of hippocampus myelin. Myelin damage is also indicated by the increased level of myelin-associated glycoprotein (MAG) that is released from damaged myelin due to fluoride intoxication.^84^ Regulation of MAG is under the control of cyclic adenosine monophosphate (cAMP) and protein kinase A (PKA) activities.^89^ Brain-derived neurotrophic factor (BDNF) and cAMP-response element-binding protein (CREB) are the downstream signaling molecules of the cAMP/PKA pathway.^90^ The above experimental study showed that fluoride intoxication decreases the mRNA expression and protein level of both CREB and BDNF suggesting altered synaptic structure and also myelin damage in the hippocampus.

### Redox imbalance

#### Nrf2 signaling pathway

Nuclear factor erythroid 2-related factor 2 (Nrf2) and Kelch-like ECH-associated protein 1 (Keap1) are associated with cellular oxidative status. An experimental study on Zebrafish by treatment with sodium fluoride revealed increased mRNA expression of Nrf2 and decreased expression of Keap1. This study also found higher mRNA expression of GST, CAT, *Nqo1* and p38 genes, which are the target of the Nrf2/Keap1 pathway and causes altered redox status. This study depicted that altered expression of such genes might be due to combat of fluoride-induced stress in fish.^91^

### Cognitive defects

#### Calpain signaling cascade

Fluoride enhances free cytosolic calcium concentration in brain tissues. Important effector molecules of intracellular calcium are calpains, which are associated with cognitive defects. Stimulation of the calpain signaling is associated with synaptic plasticity and it plays a pivotal role in learning as well as long term memory. Downstream effectors of the calpain-1 are pleckstrin homology domain leucine-rich repeat protein phosphatase 1 (PHLPP1), GTPase RhoA, and protein kinase ERK1/2. Other downstream signaling molecules are BDNF and CREB. Fluoride stimulates calpain-1 in the hippocampus cells and activates GTPase RhoA. Fluoride is also responsible for cleavage of PHLPP1 and increased phospho-ERK1/2 kinase level thereby declining the activity of ERK1/2. Activities of BDNF and CREB become also down-regulated by overexposed fluoride.^92^ Therefore, it can be concluded that fluoride alters the calpain-1 cascade and disrupts the link between the early and late long term potentiation phases thereby decreasing the cognitive capacity.

#### Effects on synaptic transmission

One important biomarker of the synaptic vesicles is synaptophysin and its concentration is correlated with cognitive impairment. One cytoskeleton-associated protein is a developmentally regulated brain protein (Dbn), which interacts with the actin filaments and plays an important role in synaptic plasticity. Microtubule-associated protein 2 (MAP2) helps to deliver neurotransmitters and maintains normal neuronal functions. The activity of glutamate receptor N-methyl d-aspartate receptor (NMDAR) is associated with synaptic transmission as well as synaptic plasticity. Fluoride decreases mRNA and protein expression of synaptophysin, MAP2, Dbn and NMDAR resulting in cognitive dysfunction and attenuated neuronal functions.^93^

#### Effects on IR signaling pathway

Insulin receptor (IR) signaling has a pivotal role in neuronal plasticity, survival of the brain, learning as well as memory.^94^ IRs are mainly distributed in the outermost layer of the olfactory bulb (OB), Hippocampal cornuammon 3 (CA3), dentate gyrus (DG) and also in cornuammon 1 (CA1) regions. Fluoride down-regulates both mRNA and protein expression of IR in OB and hippocampus which in turn affects learning along with memory ability.^95^

#### Impairment of learning and memory

Glutamate is a neurotransmitter of the excitatory synapses in the central nervous system and plays an important role in learning and memory through ionotropic and metabotropic glutamate receptors (mGluRs). mGluR5 is a type of group I mGluR which is abundant in the cerebral cortex and hippocampus and associated with synaptic plasticity, learning and memory. Fluoride decreases the mRNA and protein expression of mGluR5 in the hippocampus and cerebral cortex which in turn decreases memory and learning ability.^96^

Nicotinic acetylcholine receptors (nAChRs) are transmitter gated ion channels located in the neuronal membrane and composed of α and β subunits. To date, nine different α and three different β subunits have been identified. Fluoride decreases mRNA and protein expression of α4 and α7 subunits of nAChR but the expression of β2 subunit remains unaltered. By this process, fluoride causes neuronal dysfunction through impairment of learning and memory.^97^

#### BDNF-TrkB signaling cascade

Fluoride mediates developmental neurotoxicity by impairment of synaptogenesis through disrupting ERK1/2-mediated BDNF-TrkB signaling cascade. BDNF, the key regulator of cognitive function binds to specific receptor tyrosine kinase B (trkB) and plays fundamental roles in neuronal differentiation and survival, synaptic maturation and dendritic outgrowth. Intracellular targets of BDNF-TrkB axis are postsynaptic density protein-95 (PSD-95) and synaptophysin (SYN) which are involved in maintenance of synapses. Fluoride reduces the expression of both PSD-95 and SYN in rat hippocampus as well as in human neuroblasoma SH-SY5Y cells. Fluoride also disrupts the BDNF-TrkB axis evidenced by accumulation of BDNF and reduction of TrkB. Fluoride also increases the expression of phospho-extracellular signal regulated kinase 1 and 2 (p-ERK 1/2), while inhibition of p-ERK 1/2 attenuates the effects of fluoride. Therefore, it can be concluded that fluoride interrupts ERK1/2 mediated BDNF-TrkB signaling cascade and impairs synaptogenesis which in turn causes cognitive dysfunctions.^98^

### Altered brain function

#### Altered expressions of NCAM

Neural cell adhesion molecule (NCAM), a cell surface glycoprotein, which is expressed in the nervous system having three isoforms NCAM-120, NCAM-140 and NCAM-180 according to molecular weight, which are associated with migration of cells, axonal growth and also synaptogenesis.^99^ NCAM is also important for neuronal plasticity in brain development and controls some brain functions.^100^ Incubation of rat primary hippocampal neuron with different doses of sodium fluoride for 24 h leads to decreased mRNA expression and protein level of NCAM in a dose-dependent manner, which alter normal brain function and is responsible for neurotoxicity.^101^

#### Altered level of cytokines

Microglia are a kind of immune cell population in the central nervous system (CNS) and an important marker of activated microglia is CD11b (OX-42) expression. Fluoride increases the number of OX-42 positive cells in rat brain in a dose-dependent manner and activated microglial cells, in turn, produce some inflammatory cytokines like TNF-α, IL-1β, and IL-6. Increased level of TNF-α activates a pro-apoptotic pathway in neurons and is also associated with neurodegenerative and neurotoxic conditions.^102^

#### β-catenin mediated pathway

Fluoride dephosphorylates and activates glycogen synthase kinase 3- β (GSK-3β), which in turn activates β-catenin that triggers phosphorylation of target protein and proteasomal degradation. Targets of β-catenin are cyclin-D1 and c-myc. Cyclin-D1 has a role in cell cycle regulation and promotes neuronal proliferation while c-myc deficiency results in cell death and decrease in brain size. By deactivating these genes through the GSK-3β/β-catenin signaling pathway fluoride causes neuronal damage.^85^

#### Effects on microtubules

Two isoforms of the αβ tubulin dimers are Tubα1a and Tubβ2a which are usually co-assembled into the neuronal microtubules. Fluoride declines the expressions of both Tubα1a and Tubβ2a in the hippocampus, which demonstrates that microtubule lesion is one mode of action of fluoride toxicity that leads to neurodegenerative diseases.^103^

#### Effects on glucose utilization

Fluoride toxicity is also associated with decreased glucose utilization and other neurodegenerative changes.

The prime glucose transporter (GLUT) of mammalian blood brain barrier (BBB) is GLUT1 which transports glucose in brain and deficiency of GLUT1 in the BBB results insufficient energy supply within brain. Glial fibrillary acidic protein (GFAP) is belonging from class III intermediate filament and specific component of cytoskeletons in astrocytes during development. BDNF is another marker responsible for synaptic pasticity. Fluoride down-regulates the proteomic expression of GFAP and GLUT1 while up-regulates the expression of BDNF in cerebral cortex and hippocampus irrespective of sex of animals.^104^ Altered expression of such markers lead to developmental neurotoxicity which may also result impairment of learning and memory as well as several neurodegenerative changes including acute neuronal degeneration, brain atrophy as well as interstitial neuronal edema.

### Mitochondrial dysfunctions

#### Altered fusion and fission

Mitochondria is a dynamic organelle which incessantly remodeled by fusion and fission process which is operated in equilibrium for proper maintenance of mitochondrial morphology and function. In mammals, mitochondrial fission is required for growing and dividing cells to be populated with sufficient number of mitochondria and is mediated by fission-related protein-1 (Fis1), mitochondrial fission factor (MFF) and dynamin-related protein- 1 (Drp1). On other hand, mitochondrial fusion promotes complementation between mitochondria and is regulated by mitofusin-1 (Mfn1), mitofusin-2 (Mfn2) and optic atrophy-1 (Opa1). Impairment of mitochondrial fusion and fission results mitochondrial dysfunctions and leads to several pathological conditions including neuronal disorders.^105^

One experimental report states that fluoride up-regulates the expression of Mfn1 and Mfn2 while down-regulates the expression of Drp1 and Fis1 in human neuroblastoma SH-SY5Y cells.^105^^,^^106^ Interestingly, controversial report also exists which demonstrates that the proteomic level of Mfn1 is reduced while such level of Drp1 and Fis1 is increased in the cortical region of rat due to fluoride exposure.^107^

These findings firmly pointed out that fluoride affects mitochondrial fusion and fission in neuronal cells which lead to altered mitochondrial dynamics and brain injury.

#### Altered mitochondrial biogenesis

The expression of mitochondrial biogenesis markers which are positively correlated with children's IQ scores are peroxisome proliferator-activated receptor γ coactivator-1α (PGC-1α) and mitochondrial transcription factor A (TFAM). It has been reported that the level of such markers become decreased in peripheral blood lymphocytes of children belonging high fluoride contaminated area. Fluoride also causes decreased mitochondrial DNA content due to impairment of PGC-1α/NRF1/TFAM signaling pathway and therefore decreases mitochondrial biogenesis. Fluoride mediated decreased levels of Nrf2 and hemeoxygenase (OH-1) also support the evidence of altered mitochondrial biogenesis. These findings speculate that fluoride mediates perturbation of mitochondrial biogenesis and dynamics which in turn causes impaired neuronal development.^108^

### Cell death

#### Autophagy

A common marker of autophagy is Beclin-1, increased expression of which indicates up-regulation of autophagy and it has been reported that fluoride increases the expression of Beclin-1 in a dose-dependent manner. Increased autophagosome formation is related to an enhanced level of lipofuscin in the CA1 region^86^ of the brain. Increased lipofuscin level also enhances ROS production and^109^ interferes hormonal regulation of the nerve cells.^110^ Both of which lead to disruption of nerve cell function. Lipofuscin inhibits proteasomal pathway and then autophagy pathway compensate this for waste removal and over-activity of autophagy pathway has a negative impact on cell survival that leads to apoptosis.^111^ Fluoride also increases the protein level of Bax and decreases the level of Bcl2 in rat brain cells and promotes apoptosis.^102^

LC3-II, Atg5 and p62 are important markers responsible for autophagosome formation and degradation. Fluoride decreases the expression of LC3-II and Atg5 while level of p62 become increased in rat hippocampus as well as in SH-SY5Y cells and affects autophagy.^112^

Autophagy is also regulated by upstream signaling molecule which is mTOR and blemish of such signaling is associated with defective autophagy. Environmental factors affect the phosphorylation of mTOR that in turn hampers the phosphorylation of p70^S6K^ which is a crucial downstream factor of mTOR that ultimately affects the autophagy. Fluoride up-regulates the expression of mTOR and p70^S6K^ both in-vivo and in-vitro^112^*.* This study concludes that fluoride suppresses autophagy through mTOR/p70^S6K^ signaling pathway in neuronal cells.

#### ER stress and altered autophagic flux

When the function of endoplasmic reticulum (ER) is disrupted by external or internal stimuli then misfolded or unfolded proteins become accumulated within ER and generate ER stress. Prolonged stress response may leads to cell death. Autophagy is a kind of cell death where damaged organelles are sequestered in autophagosome and fuse with lysosome for degradation. This dynamic flow of autophagosome formation, maturation and degradation is called autophagic flux.

Hence, this equilibrium is essential for maintenance of cellular homeostasis and any alteration in this regard leads to degenerative outcomes. Interplay between the ER stress and autophagy reveals that when ER stress occur then autophagy may be triggered to maintain homeostasis by removing degraded proteins and damaged organelles. One experimental study revealed that fluoride causes ER stress and associated apoptosis as evidenced by enhanced expression of IRE1α, GRP78, cleaved caspase-12 and cleaved caspase-3 in both rat hippocampus as well as SH-SY5Y cells*.* Fluoride mediated defective autophagy was also manifested by up-regulation of Beclin1, LC3-II and p62. Cumulatively above findings firmly emphasized that fluoride causes excessive ER stress along with dysfunction of autophagic flux.^83^

#### p53 mediated pathway

One important marker of apoptosis is p53 which promotes apoptotic event by transcription dependent as well as independent pathways. In transcription dependent pathway several pro-apoptotic genes such as bax, PUMA and NOXA become activated while in transcription independent pathway, p53 translocates from cytoplasm to mitochondria and interacts with anti-apoptotic protein BCL. Both these pathways cause mitochondrial outer membrane permeabilization and release of cytochrome C which activates caspase-3. Activated caspase-3 cleaves PARP protein which is also an important marker of apoptosis. SIRT1 is a NAD^+^ dependent deacetylase which also regulates cell death and p53 is a non histone substrate of SIRT1. Fluoride up-regulates the activities of PUMA, cytochrome C, cleaved caspase 3, cleaved PARP and bax while down-regulates the level of Bcl-2 in human neuroblastoma SH-SY5Y cells. Fluoride inhibits deacetylase activity of SIRT1 and increases p53 level in SH-SY5Y cells. Therefore, it can be concluded that fluoride promotes apoptosis by inhibiting deacetylase activity of SIRT1 and promotes nuclear translocation and transcriptional activity of p53 and activates mitochondrial apoptotic pathway.^113^

#### Extrinsic apoptotic pathway

The extrinsic pathway of apoptosis depends on ligation of Fas with Fas ligand (FAS-L). Such binding activates downstream signaling molecule pro-caspase-8 and generates cleaved caspase-8 that ultimately activates caspase-3 which is a key executioner caspase. Fluoride enhances the expression of Fas, Fas-L, caspase 8 and caspase 3 in human neuroblastoma SH-SY5Y cells which suggests the occurrence of fluoride induced extrinsic apoptosis in neuronal cells.^114^

### Male reproductive disorders

**Figure 2:**
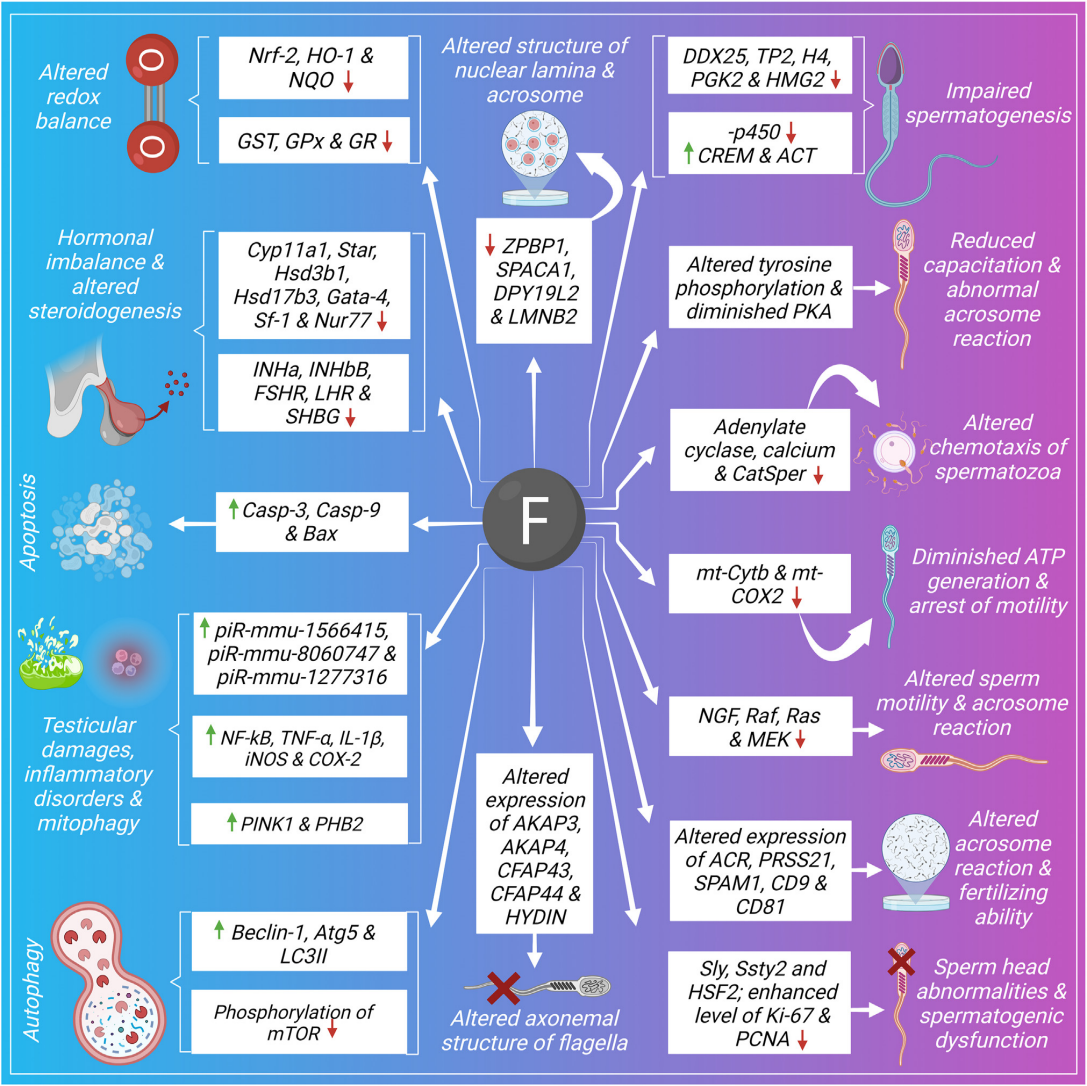
Signaling pathways of fluoride induced male reproductive toxicity.

Excessive consumption of fluoride leads to male reproductive disorders as evidenced by altered testicular as well as epididymal structure, abnormal spermatozoa status and also decreased serum testosterone level.^115^, ^116^, ^117^ Enhanced oxidative stress generation due to overproduction of ROS is a key player of fluoride mediated reprotoxicity and spermatozoa are prone to oxidative damages due to abundance of polyunsaturated fatty acids and limited antioxidant ability.^118^ Signaling pathways which are the targets of fluoride toxicity and alterations of which leads to male reproductive abnormalities are summarized here (Fig. 2).

### Altered redox balance

#### Nrf2 mediated pathway

If a cell is under any stress condition then Nrf2 dissociates from Keap1, which is a repressor of Nrf2 and translocates to the nucleus and then binds to several elements, involved in antioxidant defense such as HO-1, g-GGL, and NQO.^119^ It has been reported that upon fluoride intoxication, expression of all these proteins (Nrf2, HO-1, g-GGL, NQO) become decreased and Keap 1 expression becomes increased in rat testis. So fluoride causes testicular toxicities by oxidative stress generation through the Nrf2 signaling pathway.^75^

#### Decreased antioxidant status

It has been reported that fluoride decreases genomic and proteomic expression of glutathione related enzymes such as GST, GPx and GR in the epididymis and therefore reduces the total antioxidant capacity of the epididymis.^120^

### Altered steroidogenesis and hormonal disbalance

Key regulatory enzymes of testicular steroidogenic pathway are cholesterol side-chain cleavage enzyme (Cyp11a1), steroidogenic acute regulatory protein (Star), 3β-hydroxy dehydrogenase type I (Hsd3b1) and 17β-hydroxy dehydrogenase type III (Hsd17b3). Some transcription factors regulate the expression of these genes include GATA binding protein-4 (Gata-4), steroidogenic factor-1 (Sf-1), nerve growth factor IB (Nur77) and nuclear receptor subfamily 0 group B member 1 (Dax-1). Fluoride decreases genomic expression of Cyp11a1, Star, Hsd3b1, Hsd17b3, Gata-4, Sf-1 and Nur77 but the genomic expression of Dax-1 increased in TM3 Leydig cells. By this mechanism, fluoride affects testicular steroidogenic activities.^121^

Fluoride inhibits the expression of mRNA of inhibin alpha (*INHα*), inhibin beta-B (*INHβB*), follicle-stimulating hormone receptor (FSHR), luteinizing hormone receptor (LHR) and sex hormone-binding globulin (SHBG) in testis. FSHR and gene expression of SHBG are observed in Sertoli cells while LHR is present in Leydig cells and inhibin B regulates FSH secretion from the testis. By gene expression study it can be concluded that fluoride is responsible for functional lesions of the primary reproductive organ.^122^

### Altered testicular function

#### piRNA mediated pathway

PIWI-interacting RNAs (piRNAs) are closely associated with gametogenesis, specifically regulating genomic structure and expression during gametogenesis. Among 28 differentially expressed piRNAs, fluoride enhances the expressions of piR-mmu-1566,415, piR-mmu-8060,747 and piR-mmu-1277,316 which in turn enhance the expression of downstream target genes such as Gga2 which is associated with protein transport in the Golgi network and lysosomes; Ap4e1 and Ap1s3 which are associated with acidic hydrolysis of lysosomes and *Gla* which is associated with lysosomal signaling. Altered expressions of piRNAs and their target genes affect the lysosomal degradation pathway and cause testicular damages.^123^

#### Testicular inflammation

Regulation of testicular steroidogenesis and spermatogenesis as well as the maturation of semen are under the control of some inflammatory mediators such as NF-κB, TNF-α, interleukin-1β (IL-1β), iNOS and cyclooxygenase-2 (COX-2).^124^ All these mediators are up-regulated in case of inflammation and damage spermatogenesis. It has been reported that fluoride promotes testicular inflammation by up-regulating these genes.^125^

#### Mitophagy

Mitophagy is a dynamic and complex cellular process which regulates mitochondrial quantity and quality. It is also a kind of autophagy that has an important role in the protection of eukaryotic cells against deleterious damages and excessive mitochondrial accumulation. Among several pathways, PINK1/Parkin pathway is involved in the elimination of damaged mitochondria and hence mitophagy. PHB2 is an inner mitochondrial membrane protein and also a marker of PINK1/Parkin-mediated mitophagy in the mitochondria. Fluoride enhances mRNA and protein expression of PINK1 and PHB2 in testicular tissues and TM3 Leydig cells resulting in mitochondrial impairment as well as mitophagy in Leydig cells.^126^

### Apoptosis

#### FAS and caspase mediated pathway

Fluoride inhibits the proliferation of Leydig cells and promotes stress-induced apoptosis in a time and dose-dependent manner.^127^ Fluoride also causes enhanced expression of pro-apoptotic genes such as Caspase-3, Caspase-9 and Bax in Leydig cells and inhibits the expression of anti-apoptotic genes Bcl-2, therefore, promotes apoptosis.

Fluoride also enhances the germ cell apoptosis which was evidenced by over-expressed caspase −3 and Fas, chromatin condensation and DNA fragmentation.^128^ Enhanced level of cytochrome C, which is a mitochondrial pro-apoptotic marker released due to mitochondrial outer membrane permeabilization also supports the evidence of apoptosis.^128^

#### ER stress and unfolded protein response

Maturation and proper folding of proteins, calcium homeostasis as well as lipid biosynthesis occur in endoplasmic reticulum. Thus accumulation of misfolded or unfolded proteins in the endoplasmic reticulum leads to endoplasmic reticulum stress or ER stress and unfolded protein response (UPR). UPR signaling consists of several protein sensors such as PKR-like ER kinase (PERK), inositol-requiring protein1 (IRE1) and activating transcription factor 6 (ATF6) which suppress the protein synthesis and regulate the expression of UPR target genes which include glucose-regulated protein 78 kDa (GRP78) and a pro-apoptotic transcription factor CHOP. Activated PERK phosphorylates eukaryotic translation initiation factor 2α (eIF2α) which in turn activates CHOP. Fluoride up-regulates the ER stress markers GRP78, PERK, p-eIF2α and CHOP in Sertoli cells.^129^ Therefore, it can be concluded that fluoride promotes ER stress and UPR that ultimately leads to apoptosis in Sertoli cells.

### Autophagy

#### Impairment of autophagosome formation and degradation

Beclin 1 is an autophagy protein, which regulates autophagy initiation and autophagosome-lysosome fusion. *Atg5* and LC3 II are other proteins responsible for autophagosome formation. Fluoride promotes autophagy in primary Leydig cells by increasing the expression of mRNA and protein of these autophagy markers.^130^

Interestingly another report revealed that expression of Beclin 1 does not alter due to fluoride exposure but expression of LC3 II become enhanced in testis which indicates increased presence of autophagosome.^128^ Another autophagic substrate is p62 which selectively incorporates in autophagosome by direct binding with Lc3 and ultimately degraded within lysosome after autophagosome-lysosome fusion. Therefore, increased presence of p62 designates block of autophagosome degradation. Fluoride intoxication increases the expression of p62 in testis.^128^ Therefore, above findings firmly indicate that fluoride promotes autophagosome accumulation by inhibiting autophagosome degradation in testis.

#### mTOR signaling pathway

Mammalian targets of rapamycin (mTOR) incorporates signals that inhibit autophagy and the regulators of mTOR are AKT and AMPK. Dephosphorylation of mTOR suppresses its activity. Fluoride inhibits phosphorylation of mTOR, decreases the activity of ATK and promotes the expression of AMPK in Leydig cells. Therefore it can be concluded that fluoride enhances autophagy in Leydig cells by the mTOR signaling pathway.^130^

### Spermatozoal abnormalities

#### Impairment of spermatogenesis

Gonadotropin-regulated testicular RNA helicase (GRTH/DDX25), present in cytoplasm and nucleus of round spermatid and meiotic spermatocytes is responsible for spermatogenesis.^131^ The genes, expression of which is under the regulation of DDX25 are transitional protein-2 (TP2), H4, phosphoglycerate kinase 2 (PGK2) and HMG2 are also engaged in the maturation of sperm and regulate normal chromatoid body structure in haploid spermatogenic cells.^132^ Fluoride exposure decreases the mRNA and protein expression level of these genes and impairs spermatogenesis.^133^

Cytochrome P450 enzymes are engaged in the metabolism of xenobiotic compounds and also detoxification of the body. In the male reproductive system expression of P450 protein has been found in Leydig cells, spermatogonium and spermatocyte. It has been reported that fluoride decreases the expression of P450 in testis.^134^ cAMP-responsive element modulator (CREM) is a protein, which regulates a balance between germ cells differentiation and apoptosis in the male reproductive system.^135^ Expression of activator of CREM in testis (ACT) is coordinated with CREM during differentiation of germ cells.^136^ Fluoride increases the level of both CREM and ACT and this altered expression of these proteins may be associated with obstruction of spermatogenesis in testes.^134^

Zona pellucida binding protein 1 (ZPBP1), Sperm acrosome associated 1 (SPACA1) and dpy-19like 2 (DPY19L2) are key regulatory proteins responsible for acrosome biogenesis. Lamin B2 (LMNB2) gene encodes a protein which helps in the formation of the nuclear lamina. Fluoride decreases genomic and proteomic expression of all these genes. This study firmly suggested that fluoride can alter the ultrastructure of nuclear lamina and acrosome during spermatogenesis and hence disturbs spermatogenesis.^137^

Ki-67 and proliferating cell nuclear antigen (PCNA) are associated with proliferation as well as differentiation of spermatogonial cells in testis. Fluoride increases the expression of both the genes in spermatogenic cells in the testis and causes spermatogenic dysfunctions in the testis.^138^

#### Effects on capacitation and acrosome reaction

Previously it has been reported that late stages of capacitation include several signaling events in spermatozoa, including protein tyrosine phosphorylation, intracellular alkalinization and PKA activation.^139^ Fluoride diminishes tyrosine phosphorylation, PKA activation and also decreases intracellular calcium concentration and thereby reduces capacitation and acrosome reaction in sperm.^140^

ACR protein is a trypsin-like serine protease present on sperm acrosome and plays an important role in the progression of sperm through cumulus cells. PRSS21 is a serine protease responsible for sperm maturation in the epididymis. SPAM1 is an epididymal protein and helps spermatozoa to break cumulus cells. CD9 and CD81 are transmembrane proteins of epididymosome and promote signal transduction. Fluoride alters the expression of all these proteins and affects acrosome reaction as well as fertilizing ability of spermatozoa.^141^

#### Altered chemotaxis

Proper guidance by which sperm progress towards a chemoattractant in a concentration gradient manner secreted by oocyte and finally reaches the oocyte is called chemotaxis. After binding of chemoattractant with the sperm plasma membrane receptor, intracellular adenylate cyclase, cAMP and calcium concentration become increased. Fluoride decreases the concentration of adenylate cyclase and calcium ions in a dose and time-dependent manner. Cation channels of sperm (CatSper) is a channel protein in the flagellum, which allows calcium for entering into cells and fluoride also inhibits the expression of the CatSper 1 gene. Altered expression of these genes may reveal that fluoride affects the chemotaxis of spermatozoa.^142^

#### Arrest of spermatozoal motility

Fluoride diminishes sperm mitochondrial respiration and halts ATP production. Ubiquinol cytochrome c reductase in complex III of the electron transport chain and mt-Cytb is a subunit of this protein. The terminal protein of the electron transport chain is COX, which catalyzes the generation of water from oxygen with concomitant production of ATP. Fluoride has been known to inhibit the genomic expression of mt-Cytb and mt-COX2.^143^ This study clearly emphasized that fluoride arrests sperm motility by diminishing ATP generation through the electron transport chain.

In the MAPK pathway, nerve growth factor (NGF) is a neurotrophin family member that plays a critical role in maintaining sperm functions like sperm motility and acrosome reaction.^144^ NGF functions in concert with Ras, Raf and MEK genes and forms an NGF-Ras-Raf-MEK cascade that activates the MAPK signal pathway.^145^ Fluoride down-regulates the expression of mRNA of testicular NGF, Ras, Raf, and MEK genes and also decreases the protein expressions of NGF and MEK^146^ thereby hampering spermatozoal function.

#### Structural abnormalities

Structural integrity of spermatozoal flagella is dependent on intact 9 + 2 arrangement of the microtubule. Eleven key regulatory proteins are associated with flagellar structure among which fluoride alters the expression of five regulatory genes. These include A-kinase anchoring protein 3 (AKAP3), A-kinase anchor protein 4 (AKAP4) which are associated with fibrous sheath structure of flagellum; Cilia And Flagella Associated Protein 43 (CFAP43), Cilia And Flagella Associated Protein 44 (CFAP44) which are associated with the structural integrity of doublet microtubule and HYDIN which is associated with the core tubule of the spermatozoal flagellum.^126^ Following this mechanism fluoride alters the fibrous sheath and axonemal structure of sperm flagellum.

Sly and Ssty2 genes are located on the male-specific region of the Y chromosome and expressed during spermatogenesis. Heat Shock Transcription Factor 2 (HSF2) gene is expressed during embryogenesis in the nuclei of the meiotic and also in differentiated male germ cells. Fluoride decreases both genomic and proteomic expression of Sly, Ssty2 and HSF2 which results in enhanced sperm head abnormalities.^115^

### Female reproductive disorders

**Figure 3:**
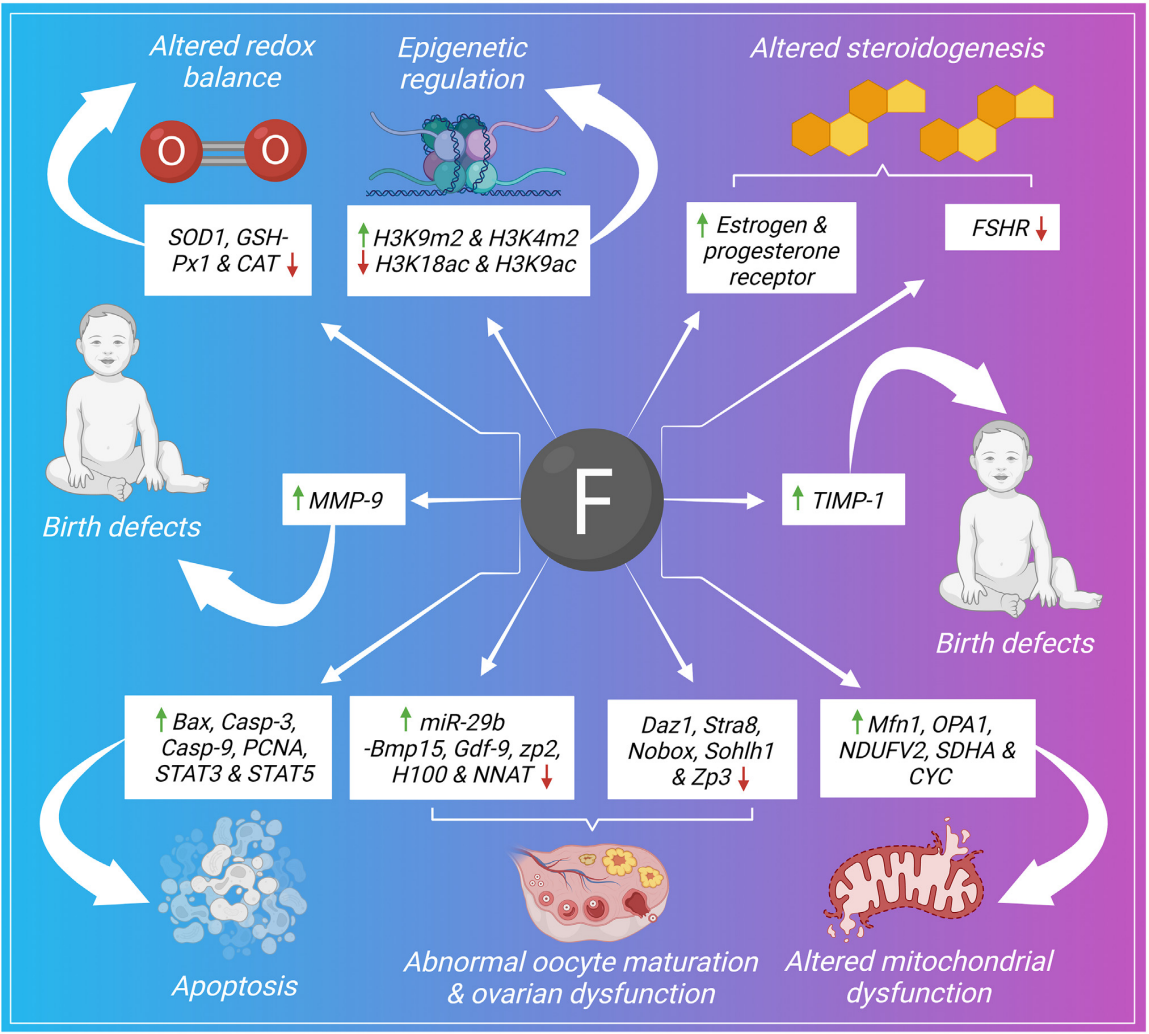
Signaling pathways of fluoride induced female reproductive toxicity.

Fluoride damages the female reproductive system markedly. Larger endometrial cells and hypertrophic endometrial glands have been reported due to overexposed fluoride.^147^ The follicular number also becomes changed in terms of increased small follicle number and decreased large follicle number. Successful fertilization rate also becomes decreased due to fluoride threat. The altered signaling pathways due to overexposed fluoride in the female reproductive system are summarized here (Fig. 3).

### Oxidative stress

It has been reported that fluoride diminishes the level of mRNA expression of some potent antioxidant markers like SOD1, GSH-Px1 and CAT in ovarian tissues.^148^

### Altered steroidogenesis

Fluoride decreases serum estrogen and progesterone levels but interestingly western blot analysis revealed that it enhances protein expression of these two hormone receptors. It has been predicted that fluoride might interfere with the hormone-binding domain of estrogen receptor (ERα) and inhibit the binding of estrogen to the receptor. It has been also assumed that fluoride, by interrupting the expression of these hormone receptors may create adverse conditions on embryo implantation and as well as endometrial receptivity.^147^

Fluoride down-regulates the expression of follicle-stimulating hormone receptor (FSHR).^147^ FSHR is a G protein-coupled receptor (GPCR) and fluoride alters the conformation of G protein,^149^ which regulates the level of secondary messengers (cAMP and Ca^2+^).

### Altered mitochondrial function

#### Effect on granulosa cell

Fluoride up-regulates the expression of Mfn1 and OPA1, which are responsible for mitochondrial fusion in granulosa cells of the ovary. Altered expression of these proteins due to fluoride exposure cause abnormality in mitochondrial fusion in ovarian granulosa cells.^25^

#### Effects on follicle and oocyte development

Fluoride enhances the expression of NDUFV2, SDHA and CYC, which are respiratory chain complexes in mitochondria and participate in intracellular oxidative phosphorylation and as well as ATP synthesis.^25^ So it can be hypothesized that fluoride causes damage to the mitochondrial respiratory chain and that leads to mitochondrial dysfunction of granulosa cells, which affects follicular development and also the potential of oocytes development.

### Ovarian dysfunction

#### miRNA mediated pathway

miRNAs are small non-coding RNAs which negatively regulate mRNA expression through cleaving of target mRNA or by repressing translation. miR-29 b is a type of miRNA which is responsible for female gonadal development. Fluoride up-regulates the expression of miR-29 b in granulosa cells and leads to ovarian dysfunction.^150^

#### Effects on formation and maturation of oocyte

It has been reported that several germline-specific genes are associated with oocytes maturation in the ovary which includes DAZL (maintenance, differentiation and proliferation of primordial germ cells), Stra8 (progression of meiosis in the ovary), Nobox (folliculogenesis), Sohlh1 (oocytes differentiation) and Zp3 (formation of zonapellucida). Fluoride decreases the expression of all these genes in the ovary and affects the formation and fertilization of mature oocytes.^151^

Activities of some genes are associated with oocytes growth and acrosome reaction which are Bmp15 (follicular development and oocytes maturation), Gdf-9 (growth and differentiation of granulosa cells), zp2 (acrosome reaction and prevention of polyspermy) and H1oo (maturation of oocytes). Fluoride mediated down-regulation of these genes results in abnormal oocytes maturation.^151^

NNAT gene is responsible for oocytes maturation by glucose transportation in oocytes and methylation causes inactivation of this gene. Fluoride causes hypermethylation of this gene and results its decreased expression which in turn affects the quality of oocytes by diminishing glucose transport.^152^

### Apoptosis

Ovarian development and follicle formation in the ovary depend on STAT3 and STAT5 protein which are members of the JNK/STAT signaling pathway and altered expressions of these proteins are associated with follicular atresia and ovarian cysts formation.^153^ An experimental study revealed that fluoride intoxication causes decreased expression of JNK, which might be due to fluoride-induced oxidative stress and enhanced expression of STAT3 and STAT5, which was hypothesized as compensatory response upon fluoride-induced damage of granulosa cells and also follicular dysplasia in the ovary.^154^ The proliferation of cells and regulation of cell fate are under the control of CycD-Cdk4 and CycD-Cdk2, which act via the STAT family.^155^ It has been reported that fluoride exposure causes over-expressions of CDK2 and CDK4, which might be due to enhanced levels of STAT3 and STAT5 and all these events result in apoptosis of granulosa cells and follicular dysplasia in the ovary.^154^

Fluoride also increases the expression of PCNA, which is a cell proliferation marker and enhanced expression of this marker reflects DNA damage and apoptosis of granulosa cells.^154^

Protein expression of some pro-apoptotic markers like Bax, caspase-3 and caspase-9 have also been reported to elevate upon fluoride exposure in the ovary.^148^

#### Birth defect

Pregnancy is linked with embryo implantation, which is under the control of the MMP-9/TIMP-1 system.^156^ MMP-9 is a member of MMPs and TIMP-1 is belonging to TIMPS; a combination of these two control invasion of trophoblast and also angiogenesis at the time of embryo implantation.^157^ Fluoride has been known to up-regulate the expression of MMP-9 and TIMP-1 and that leads to a decreased litter size of the female mice.^158^

### Epigenetic regulation

DNA methylation and histone H3 modifications are epigenetic regulation of gene expression. Histone H3 modification includes H3K9m2, which is a repressive mark while H3K9ac and H3K4m2 are active marks. Fluoride enhances the genomic and proteomic expression of H3K9m2 and H3K4m2 in the embryo but the expression of H3K9ac remain unaltered. Therefore it can be speculated that fluoride affects epigenetic modification-related genes and hampers methylation pattern as well as expression of developmentally related genes of the embryo.^159^

Oogenesis is under the control of DNA methylation and histone acetylation because 5-methylcytosine in the mammalian genome is required for normal development while histone acetylation is associated with transcriptional activation. Fluoride down-regulates the expression of histone acetyl transferases (H3K18ac and H3K9ac) in mature oocytes thereby hampering histone acetylation while DNA methylation patterns remain unaffected.^151^ Hence it can be concluded that fluoride affects germline line development through alteration of epigenetic regulation.

### Immune system disorders

**Figure 4:**
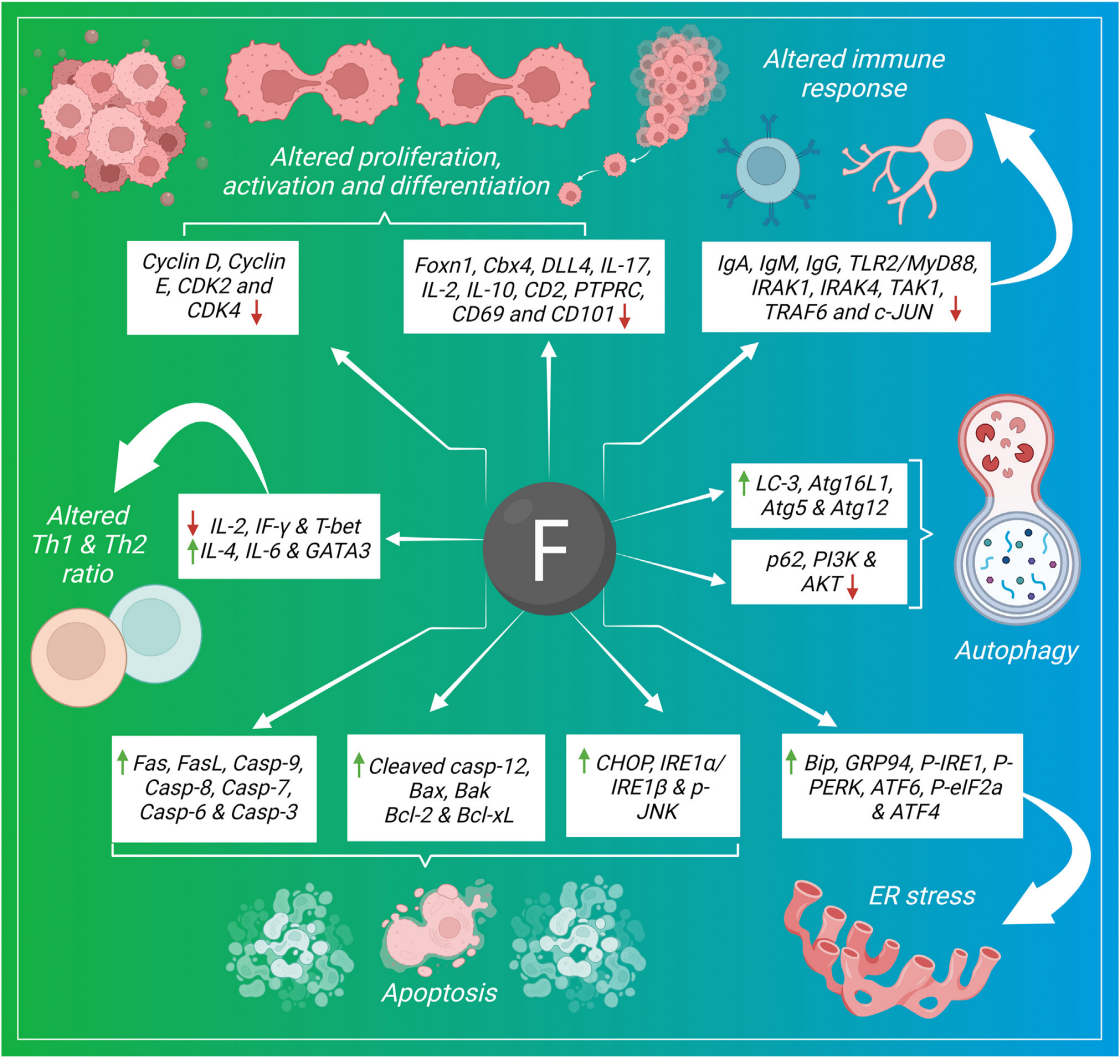
Signaling pathways of fluoride induced immune system disorders.

Fluoride causes functional impairment of the immune system in both animal models and human beings. Fluoride mediated adverse effects have been reported in the spleen, thymus, bursa of Fabricius and cecal tonsil by means of structural changes, oxidative stress and apoptosis.^160^ The mechanistic approach of fluoride toxicity in the immune system through altered signaling cascades is summarized here (Fig. 4).

### ER stress

Several conditions disturb the functions of the endoplasmic reticulum, which are collectively called ER stress and it has been reported that fluoride causes apoptosis of the spleen through ER stress pathway both *in-vivo* and *in-vitro*. BiP and GRP94 are chaperone proteins in the ER lumen and promote the folding of nascent proteins^161^ and expressions of these proteins become up-regulated upon ER stress. Under stress conditions in ER, a signaling pathway is activated in cells known as UPR following activation of several proteins like PERK,^162^ IRE1,^163^ ATF6.^164^ Upon activation of PERK, phosphorylation of eIF2α occurs and finally, ATF4 translation is promoted.^165^ An experimental study revealed that sodium fluoride enhances the protein expression of BiP, GRP94, P-IRE1, P-PERK, ATF6, P-eIF2a and ATF4 both *in-vivo* and *in-vitro* in the spleen, which indicates fluoride-induced ER stress in the spleen.^166^

### Altered ratio and proliferation of lymphocytes

Fluoride has been shown to disturb the balance ratio of Th1/Th2 cells in the spleen by altering various cytokine levels and transcription factors. IL-2 and IFN-γ, which are characteristic of Th1 cells were found to decrease along with expression of transcription factor T-bet, cumulatively leading to a decrease in Th1 cells. Alongside fluoride increases IL-4, Il-6 and transcription factor GATA3 which is responsible for the expression of Th2 cells. So the Th1/Th2 ratio is decreased under fluoride threat.^167^

Cell cycle progression from G1 to S phase in under the control of cyclin D-dependent kinases, which include Cdk4 and Cdk6 that bind to cyclin D and also under the control of Cdk2 binding to cyclin E or A.^168^ Experimentally it has been proved that sodium fluoride decreases the protein expression of cyclin D, cyclin E, CDK2 and CDK4 in the spleen, which demonstrated that fluoride intoxication inhibits proliferation of splenic lymphocytes, represented by reduced activation and proliferation of splenic B and T lymphocytes.^169^

A Th17 cytokine expression array in C2C12 cells revealed that fluoride alters many cytokine expression profile, including the IL-17 A and Il-17 F pathways, leading to altered cytokine–cytokine receptor binding and Th17 cell differentiation.^170^ In a study to find out the effects of fluoride on T cell expression and modulation of activity, it was found that fluoride decreases the expression of genes related to T cell proliferation and differentiation, namely Foxn1, Cbx4, DLL4 and IL-17. Due to decreased expressions of IL-2 and IL-10, there was decreased in CD4^+^ and CD8^+^ T cells population in the thymus. Several other genes related to T cell function like CD2, PTPRC, CD69 and CD101 were found to decrease in fluoride-treated conditions.^171^

### Altered immune response

**Effects on humoral immune response**

Other *in-vitro* studies with splenic lymphocyte cultures prepared from male ICR rats went in acceptance with the previous results. Increase in IL-2, IL-6, TNF-α, IFN-γ and TGF-β significantly, decrease in CD3^+^, CD3^+^/CD4^+^ and CD3^+^/CD8^+^ T cells and CD19^+^ B cells showed suppressive effects of fluoride on lymphocyte maturation, differentiation and cell-mediated immune response. Along with cellular immunity, fluoride also shows a marked decrease in humoral immune responses. Along with reduced CD19^+^ B cells, there was seen a significant decrease in the expression of IgA, IgM and IgG after treatment with fluoride at 24 and 48 mg/kg for 21 and 42 days.^160^

**Effects on innate immunity**

Innate immunity is the first-line defense against invading pathogens and this recognition is achieved via cell surface receptor, known as toll-like-receptor (TLR).^172^ The downstream signaling of TLR pathway involves myeloid differentiation primary response protein 88 (MyD88), and IL-1-receptor-associated kinases (IRAK) 1 and 4, transforming growth factor-β activated kinase 1 (TAK1), c-Jun and TNF receptor-associated factor 6 (TRAF6), which finally activate the transcription factor nuclear factor-kB (NF-kB).^173^ It has been reported that sodium fluoride inactivates TLR2/MyD88 signaling pathway, which is characterized by decreased expression of TLR2/MyD88, IRAK1, IRAK4, TAK1, TRAF6 and c-JUN alter the expression of IL-2, IL-4, IL-6, IL-8, IL-10, TNF-α, IFN-γ and transforming growth factor-β (TGF-β), that attenuates the innate immunity.^174^

### Apoptosis

#### ER stress

Severe ER stress for a long period of time results in apoptosis.^175^ The first apoptotic pathway of ER stress is the activation of CHOP, which is a stress-induced pro-apoptotic gene; overexpression of this gene leads to apoptosis.^176^ Though the level of expression of this protein under normal physiological conditions is low it has been reported that upon fluoride threat, the expression of this protein becomes elevated in the spleen and leads to apoptosis.^166^

Another apoptotic pathway by ER stress is the activation of the JNK pathway and this pathway is activated upon elevated expression of IRE1α or IRE1β.^177^ Activated IRE1s recruits TRAF and activates it, which in turn triggers the activity of JNK and leads to apoptosis.^177^ Fluoride increases the expression of p-JNK both *in-vivo* and *in-vitro* and promotes apoptosis.^166^

During ER stress, the cytosolic domain of IRE1 takes on tumor necrosis factor (TNF) receptor-associated factor 2 (TRAF2) that interacts with Caspase-12 and activates it and finally promotes apoptosis.^178^ Fluoride also enhances the expression of cleaved-caspase 12 and promotes apoptosis.^166^

#### Extrinsic and intrinsic pathways

Intracellular apoptotic signaling cascade is under the control of Bcl-2 family members, which include both pro-apoptotic (Bax and Bak) and anti-apoptotic (Bcl-2, Bcl-xL) proteins.^179^ An experimental study demonstrated that sodium fluoride down-regulates the expression of Bcl-2 and Bcl-xL while up-regulates the expression of Bax and Bak in lymphocytes and the ratio of Bax/Bcl-2 was also elevated along with elevated p53,^180^ which suggests fluoride-induced apoptosis of lymphocytes.^166^

After receiving a death signal, cytochrome C is released and Caspase-9 is activated, which finally activate caspase-3, -6 and -7 followed by cleaving of cellular proteins and apoptosis.^181^ In the extrinsic apoptotic pathway Fas and Fas ligand (FasL) interact and activates Caspase 8, which further activates caspase3, 6 and 7 that cause apoptosis.^182^ Fluoride has been known to upregulate the protein expression of Fas, FasL, caspase-9, caspase-8, caspase-7, caspase-6 and caspase-3 in lymphocytes,^166^ which suggests fluoride also causes apoptosis through death receptor-mediated pathway.

#### Autophagy

LC3 protein acts as an autophagosome marker in mammals and after synthesized it is cleaved to LC3 I and then to LC3 II, which is associated with autophagosome membrane and autophagosome formation.^183^ An experimental study proved that sodium fluoride enhances the expression of both mRNA and protein levels of LC3 in the spleen, which suggests fluoride induces autophagic activity and the formation of autophagosome in the spleen.^184^

P62 protein is an autophagy regulator and when autophagy is induced then the expression of p62 is inhibited but autophagy is restrained then p62 become accumulated.^185^ Bclin1 is another protein, which promotes autophagy by fusion of autophagosome-lysosome via interacting with LC3-PE and Atg12-Atg5 complexes.^186^ Experimentally it has been proved that fluoride promotes autophagy by diminishing mRNA and protein expression of p62 while enhancing the expression of Beclin1 in the spleen.^184^

Atg16L1 protein forms an autophagy complex with two other proteins Atg5 and Atg12 that promote the formation of autophagosomes.^187^ Fluoride enhances the expression of both mRNA and protein of Atg16L1, Atg5 and Atg12 of the spleen and indicates that splenocytes are prone to autophagy upon fluoride threat.^184^

mTOR activity is negatively co-related with autophagy because it has been reported that mTOR integrates signaling pathway, which inhibits autophagy via thePI3K/Akt pathway.^188^ An experimental study demonstrated that fluoride down-regulates the expression of PI3 and AKT that indicates suppression of mTOR signaling and enhance autophagy.^184^

## Conclusion

It can be concluded that fluoride can alter the homeostasis of physiological systems through alteration of cellular redox status which is the foremost detrimental consequence due to overexposed fluoride. This can lead to subsequent disadvantageous conclusions including DNA damage, autophagy, apoptosis, mitochondrial dysfunction, mitophagy, cell cycle arrest and other organ-specific disorders. Although some fates of fluoride toxicity are common in different organs they may follow diverse signaling cascades in most cases.

## Author contributions

Priyankar Pal: Literature survey, primary draft. Niraj Kumar Jha: Illustrations and Revision. Saurabh Kumar Jha: Revision. Debankur Pal: Literature survey, primary draft. Uttpal Anand: Revision and expert opinion. Abhijit Dey: Guidance, revision and finalization. Prabir Kumar Mukhopadhyay: Conceptualization, guidance, finalization and overallmonitoring. All authors have read and approved the final version of the manuscript for submission to this journal.

## Conflict of interests

The authors declare no conflict of interests.
